# Controlled Release of Hydrophilic Active Agent from Textile Using Crosslinked Polyvinyl Alcohol Coatings

**DOI:** 10.3390/jfb16060216

**Published:** 2025-06-10

**Authors:** Limor Mizrahi, Rotem Kelman, Efrat Shtriker, David Meridor, Dror Cohen, Meital Portugal-Cohen, Elizabeth Amir

**Affiliations:** 1 Department of Polymer Materials Engineering, Shenkar College, Ramat-Gan 5252626, Israel; 2 AHAVA Dead Sea Laboratories Ltd., 1 Arava, Airport City 7019900, Israel

**Keywords:** polyvinyl alcohol, sustained release, allantoin, nonwoven fabric, polyacrylic acid, coating

## Abstract

Functional fabrics embedded with active materials that can be released in a controlled manner upon external triggering have been explored for biomedical and cosmetic applications. This study introduces a method for the fabrication of nonwoven fabrics coated with crosslinked polyvinyl alcohol (PVA) for in situ encapsulation and controlled release of hydrophilic active agent, allantoin. Two types of crosslinked coatings were examined using citric acid (CA) or polyacrylic acid (PAA) as crosslinkers. Based on gel content, differential scanning calorimetry (DSC) and dynamic mechanical analysis (DMA) analyses PVA:CA coatings exhibited a higher crosslinking density compared to PVA:PAA systems. Swelling behavior was measured at 62% after 30 min for PVA:PAA 7:3 films and 36% after 60 min for PVA:CA 7:3 crosslinked films. The release of allantoin from the coated fabrics was influenced by the coating thickness (250–330 µm), the formulation viscosity (8–250 cP), allantoin content (1.2–4.2 mg) and the molecular weight between crosslinks (M_C_) 1,000,000–494 g/mol. PVA:CA 7:3 coating allowed the controlled release of 97% allantoin over 8 h, whereas PVA:PAA 7:3 coating exhibited a more prolonged release profile, with 96% of allantoin released over 20 h. Kinetic analyses of the release profiles revealed a good agreement with zero-order release.

## 1. Introduction

For over 4000 years, since the first recorded use of sutures, textiles have been explored for novel applications across various fields, including medicine and cosmetics [1,2,3]. Medical textiles range from traditional items such as surgical sutures, bandages, and surgical gowns to more advanced materials like textile-based implants and extracorporeal devices, such as artificial kidneys [4] and bio-functional textiles [5]. Bio-functional textiles represent a new frontier in textile innovation, serving as delivery systems for cosmetic or pharmaceutical substances, with direct skin contact. In recent years, extensive research has focused on development of bio-functional textiles for skin applications, particularly for controlled release of therapeutic or cosmetic compounds and the absorption of substances from the skin [6,7,8,9]. The combination of advanced pharmaceutical carriers with textile enhances active molecules penetration while simultaneously regulating drug release for both topical and transdermal therapies [10,11,12]. In dermatology and cosmetics, maintaining effective contact between active agents and the skin is one of the key aspects of effective therapy [13,14,15,16,17]. While semi-solid products such as creams, gels, and ointments demonstrate sustained release, they often fail to maintain extended skin contact. In recent years, bio-functional textiles with controlled drug-release ability have attracted significant research interest due to their strong potential to overcome the need for frequent applications while ensuring prolonged skin contact and sustained release of active agents [18]. The literature volume in the field of targeted delivery of pharmaceutical reagents from textiles has rapidly increased during the past decade, and a wide variety of crosslinked coatings and therapeutic agents are examined for numerous applications. The general strategy for fabricating bio-functional textiles for prolonged delivery of active agents involves incorporating active small molecules into a crosslinked polymer network, which serves as a coating for various types of fabrics. Using this strategy, hydrogel coatings based on crosslinked chitosan and PVA have been reported for the prolonged release of paracetamol [19], cephalexin and curcumin [20,21] from silk and cotton fabrics, respectively. In addition, silane-coated cotton fabrics, prepared using a sol–gel process, were introduced for controlled release N-palmitoyl-(4-nitro-aphenyl)-amine [22] or tetracycline hydrochloride [23]. Cotton fabrics functionalized with alginate and poly-*β*-aminoester-based crosslinked coatings were introduced for controlled release of tetracycline and levofloxacin [24], and azulene [25].

Although the demand for new technologies for the applications requiring targeted delivery of pharmaceutical reagents from textiles is similar for both hydrophobic and hydrophilic active materials, the vast majority of sustained delivery systems reported in the literature are designed for hydrophobic carriers. While the release rate of hydrophobic molecules is mostly controlled by the rate of water penetration into the crosslinked polymer coating, the high dissolution rate of hydrophilic active agents due to their inherent aqueous solubility presents a unique challenge [26].

It has been shown that the sustained release of hydrophilic reagents from fabrics for several hours and even can be realized by polymer coatings with high crosslinking density and thickness, such as hydrogels based on alginate/poly(ethylene glycol) diacrylate/poly(*N*-isopropylacrylamide) [27], chitosan/salicylaldehyde/silver nanoparticles [28], wax [29], and electrospun nanofibers [30]. However, such systems are relatively rare and there is a strong need for developing new biocompatible coating formulations containing a balanced crosslinking density, thickness, and extensive intermolecular interactions that allow sustaining the release of hydrophilic carriers.

Allantoin is a natural compound and final product of purine catabolism in humans. It can also be extracted from plants and grains or chemically synthesized through the oxidation of uric acid [31,32,33]. Due to its numerous skin benefits, including anti-inflammatory properties, protective effects, and promotion of cell proliferation, allantoin is widely used in the treatment of various skin conditions, such as ulcers, acne, and psoriasis. It is also commonly found in oral hygiene products and skincare formulations [33,34,35,36,37,38].

PVA is a biocompatible polymer widely used for encapsulating active agents in drug release, tissue engineering, cosmetic, and biomedical applications. PVA has been explored for the fabrication of facial masks, scaffolds for cell culture, contact lenses, wound dressings, and transdermal drug delivery [39,40,41,42,43,44,45,46,47,48,49,50,51]. It is also considered a non-toxic polymer with an LD50 (>20 g/kg) for oral administration in rats and mice [52].

Citric acid is a biocompatible and non-toxic natural material widely used in cosmetic applications as a pH regulator, chelating agent, fragrance ingredient, antibacterial agent, and food additive [53,54,55,56,57]. Polyacrylic acid is a non-toxic, biocompatible polymer that plays an important role in the production of hygiene products, detergents, wastewater treatment chemicals, cosmetics, and more [58,59,60,61].

Coatings based on PVA, CA, and PAA crosslinked via chemical and physical bonds, have strong potential to sustain the release of allantion from the crosslinked polymer matrix by creating strong intermolecular bonding interactions with free hydroxyl and carboxylic acid moieties of the coating. In addition, the crosslinking degree of such coatings can be tuned by varying chemical and physical crosslinking bonds via the crosslinker concentration in the aqueous coating formulation and adjusting the curing conditions of the solid coatings.

Nonwoven fabrics are widely used for the production of skincare products such as bandages, facial masks, wipes, and diapers. A variety of such skincare products use thin Spunbond polyethylene and polypropylene nonwoven fabrics containing filaments of thermoplastic polymer randomly bonded together to create a web-like structure.

To the best of our knowledge, an efficient strategy for reducing the dissolution rate of hydrophilic carriers, such as allantoin, incorporated into crosslinked PVA coated fabrics via extensive intermolecular interactions balanced with crosslinking degree and thickness has not been reported yet.

Herein, we describe a strategy for in situ functionalization of nonwoven fabrics using crosslinked PVA-based coatings as reservoirs for encapsulation and controlled release of hydrophilic active agent–allantoin. Four types of crosslinked coating formulations were developed using either citric acid or polyacrylic acid as crosslinkers, using a PVA-crosslinker weight ratio of 9:1 and 7:3 for each crosslinker. The coatings were characterized using DSC, DMA, gel content, and swelling measurements. Aqueous coating formulations containing allantoin were applied on polyethylene nonwoven fabrics via deep coating, followed curing. The coated fabrics were examined for topical release of allantoin using the vertical Franz Diffusion Cell method, and the release profiles were analyzed using four kinetic models.

## 2. Materials and Methods

### 2.1. Materials

Polyethylene nonwoven fabric (22 GSM) was obtained from Shalag Company, Qiryat Shemona, Israel. Polyvinyl alcohol with a molecular weight of 30–50 kDa and a hydrolysis degree of 98–99%, Citric acid monohydrate, and poly(acrylic acid) with a molecular weight of 450 kDa were purchased from Sigma Aldrich, Rehovot, Israel. Ethanol was procured from Biolab, Jerusalem, Israel. Cellulose membranes with a molecular weight cutoff of 12 kDa were sourced from Sigma Aldrich, Israel. Allantoin was generously provided by AHAVA company, Mitzpe Ramon, Israel.

### 2.2. Preparation of Coating Formulations

PVA:CA 9:1 formulation: A 4.5 g solution of PVA (5 wt%) in 90 mL distilled water (DW) was prepared by vigorous stirring at 90 °C for 30 min, followed by cooling to room temperature (RT). Subsequently, a 0.5 g solution of CA (10 wt%) in 5 mL distilled water was added to the PVA solution to achieve a total concentration of 5 wt%. Allantoin (4.7 mmol, 0.75 wt%) was incorporated into the formulation at 50 °C, and 0.75 g and stirred until fully dissolved.

PVA:CA 7:3 formulation: A 3.5 g solution of PVA (3.9 wt%) in 90 mL distilled water was prepared by vigorously stirring at 90 °C for 30 min, followed by cooling to RT. Then, a 1.5 g solution of CA (30 wt%) in 5 mL distilled water was added to the PVA solution to achieve a total concentration of 5 wt%. Allantoin (4.7 mmol, 0.75 wt%) was incorporated into the formulation at 50 °C, and 0.75 g and stirred until fully dissolved.

PVA:PAA 9:1 formulation: A 4.5 g solution of PVA (10 wt%) in 45 mL DW was prepared by vigorous stirring at 90 °C for 30 min, followed by cooling to RT. Next, a 0.5 g solution of PAA (1 wt%) in 50 g ethanol was prepared by vigorous stirring at 70 °C for 30 min. The PAA solution was added dropwise to the PVA solution under constant stirring at 70 °C to achieve a total concentration of 5 wt%. Allantoin (4.7 mmol, 0.75 wt%) was incorporated into the formulation at 50 °C, and 0.75 g and stirred until fully dissolved.

PVA:PAA 7:3 formulation: A 3.5 g solution of PVA (7.8 wt%) in 45 mL DW was prepared by vigorous stirring at 90 °C for 30 min, followed by cooling to RT. Then, a 1.5 g solution of PAA (3 wt%) in 50 g of ethanol was prepared by vigorous stirring at 70 °C for 30 min. The PAA solution was added dropwise to the PVA solution under constant stirring at 70 °C to achieve a total concentration of 5 wt%. Allantoin (4.7 mmol, 0.75 wt%) was incorporated into the formulation at 50 °C, and 0.75 g and stirred until fully dissolved.

### 2.3. Procedure for the Fabrication of Crosslinked Thin Films and Coated Fabrics

Crosslinked thin films were prepared by pouring the coating formulations into silicon mold (2 × 2 cm^2^), followed by drying at RT for 1–2 days. Subsequently, the films were thermally cured at 130 °C for 2 h (for the PVA:CA formulations) and at 140 °C for 40 min (for the PVA:PAA formulations).

Polyethylene nonwoven fabrics (5 × 5 cm^2^) were immersed in aqueous solutions of PVA:CA/allantoin or PVA:PAA/allantoin for 5 s. The coated fabrics were dried at room temperature overnight, followed by thermal curing as described above.

### 2.4. Characterization Methods

#### 2.4.1. Fourier Transform Infrared Spectroscopy (FTIR-ATR)

FTIR spectra were recorded on a Bruker (Billerica, MA, USA) Alpha-t FTIR-ATR spectrometer within a range of 300 to 4000 cm^−1^, with a resolution of 2 cm^−1^ and 120 scans. FTIR was employed to detect any changes in the bonds resulting from variations in the esterification of the crosslinkers.

#### 2.4.2. Scanning Electron Microscopy (SEM)

Scanning electron microscopy photographs were captured using a JEOL Ltd. (Tokyo, Japan) JSM-IT200 InTouchScope™ at magnifications of X120 and X180 to examine the surfaces of both the unmodified and coated fabrics. This facilitated the observation of coating and allantoin accumulation on the fabric. The samples were coated by sputter coater (SC7620, Quorum Technologies, Laughton, UK).

#### 2.4.3. Water Contact Angle Analysis (WCA)

The wetting properties of the coated fabrics were investigated using a contact angle analyzer (OCA20, Data Physics, Riverside, CA, USA). Prior to the measurement, the fabrics were dried in a vacuum oven. The reported water contact angle values represent the averages of results obtained from five different locations on both sides of each fabric, using 5 μL Millipore water droplets.

#### 2.4.4. Swelling Test

To investigate the swelling characteristics of the crosslinked PVA films in water, rectangular-shaped samples (2 × 2 cm^2^) were dried at RT overnight, followed by curing, and then allowed to swell in water at room temperature. Once swollen, the film was removed from the water and quickly blotted with absorbent paper to remove excess surface water. It was then weighed and placed back into the same bath. The relative water uptake was measured at various time intervals until a constant weight was achieved for each sample. This weight was used to calculate the equilibrium water uptake of the hydrogel films (S) using Equation (1):

(1) $$S=\frac{\left(W_{S}-W_{D}\right)}{W_{D}}$$

where *W_S_* and *W_D_* represent the weights of the sample in the swollen and dry state, respectively.

#### 2.4.5. Gel Content

To investigate the degree of crosslinking in the crosslinked PVA-based thin films, rectangular-shaped samples (2 × 2 cm^2^) were dried at RT overnight and thermally cured as described above. Subsequently, the films were immersed in water at 90 °C for 24 h to induce dissolution of unreacted PVA and crosslinker. Following this, the films were dried at 100 °C for 12 h, and the % gel content was calculated using the following Equation (2):

(2) $$\%Gel=\frac{\left(W_{I}-W_{f}\right)}{W_{I}}$$

where *W_I_* represents the initial weight of the sample, and *W_f_* represents its weight after immersion and drying.

#### 2.4.6. Differential Scanning Calorimetry

A TA Differential Scanning Calorimeter Q200 with a RCS40 refrigerated cooling system (Thermo Fisher Scientific, Hillsboro, OR, USA) was employed to obtain glass transition temperatures (T_g_) of non-crosslinked and crosslinked polymer films. DSC thermograms were recorded between −50 and 250 °C, with a heating rate of 10 °C/min. The T_g_ values were determined as the temperatures corresponding to the midpoint of the increment in specific heat capacity (ΔC_p_) during the transitions.

#### 2.4.7. Dynamic Mechanical Analysis

Dynamic Mechanical Analysis was conducted using a TA Dynamic Mechanical Analyzer Q800 V21.3 Build 96 (TA Instruments, New Castle, DA, USA). The tests were performed within a temperature range of 25–240 °C under constant stress, with a frequency of 1 Hz and a heating rate of 5 °C/min. A rectangular shaped crosslinked film samples measuring 2.2 mm in width, 30 mm in length, and 0.2 mm in thickness were used for the DMA. The viscoelastic properties of the cured films were analyzed to determine the dimensions of the polymeric network.

According to rubber elasticity theory, when T > T_g_, at low frequencies, the storage modulus (E′) reaches a plateau. Using Flory’s rubber elasticity theory, Equation (3) was employed to calculate the average molecular weight between crosslinks (M_C_), which is a measure of the polymer network structure [62,63]. The storage modulus E’ of the films was measured at a T_g_ of 50 °C.

Equation (3):

(3) $$M_{C}=\frac{3RT\rho }{E^{\prime }}$$

R—gas constant
T—temperature [K]
ρ—material density [g/mL]
E′—storage modulus [Mpa]

Material density was measured using Archimedes’ principle, employing acetone as a poor solvent. Triplicate samples of polymer films were weighed twice: once on an elevated platform under atmospheric pressure and once in acetone, a poor solvent with a known density of 0.784 g/mL [64].

The material’s density is calculated using Equation (4):

(4) $$\rho_{polymer}=\frac{m_{polymer}}{V_{polymer}}=\frac{m_{polymer}\rho_{b}}{{\left(m_{w}-m_{m}\right)-\rho }_{b}}$$

$\rho_{polymer}$—polymer density
$m_{polymer}$—polymer mass
$V_{polymer}$—polymer volume
$\rho_{b}$—buoyant density
$m_{w}-m_{m}$—mass difference between the polymer weighed in the air and in the buoyant.

### 2.5. Release Studies

Allantoin release properties from the coated fabrics were studied using the vertical Franz Diffusion Cell method. Fabric samples were positioned between the two chambers of the Franz cell. The bottom chamber was filled with deionized water, stirred magnetically, and covered by a cellulose membrane (Mw = 14 kDa) [65,66]. The fabric sample was placed over the cellulose membrane and covered with a polyethylene sheet to ensure complete sample wetting. During the release experiment, allantoin diffused from the fabric into the water through the membrane. At each time interval, 2 mL of the solution was withdrawn and replaced with 2 mL of distilled water to maintain the vessel volume. The withdrawn sample was diluted with 1 mL of 0.1 M NaOH to a final volume of 3 mL. The release of allantoin was monitored using UV-Vis measurements at a maximum absorbance of 235 nm, using a UV–1900i SHIMADZU spectrophotometer (SHIMADZU Corporation, Kyoto, Japan). The absorption data were fitted to a calibration curve (Figure S1). Following the 24 h release test, the fabrics were extracted using 25 mL of deionized water to determine the residual allantoin concentration in the fabric. The amount of allantoin in the extracts was quantified using the same method as for the release samples. The release profiles were compared to the release of allantoin from a PE fabric without a crosslinked PVA coating, which served as a reference system.

#### Kinetic Models for the Allantoin Release from the Coated Fabrics

The drug release profiles were analyzed using a semi-empirical Ritger–Peppas Equation applicable to one-dimensional drug release from thin polymer films [67,68]. This Equation is derived from a solution to Fick’s law for diffusional transport of solute under sink conditions and is expressed as Equation (5).

Equation (5): Ritger–Peppas

(5) $$\frac{M_{t}}{M_{\infty }}=Kt^{n}$$

$\frac{M_{t}}{M_{\infty }}$—fractional drug release at time t
K—release constant
n—diffusional exponent, indicative of Fickian or non-Fickian solute transport (n = 0.5 for Fickian, 0.5 < n < 1 for non-Fickian, n = 1 for anomalous solute transports). The Ritger–Peppas equation is valid for the first 60% of drug release.

The release mechanisms derived from the Ritger–Peppas Equation for Type II transport and Fickian transport correspond to zero-order and Higuchi models, respectively. In zero-order release systems, the active reagent is released at a constant rate, independent of its concentration within the system (Equation (6)).

Equation (6): Zero order drug release model

(6) $$\frac{M_{t}}{M_{\infty }}=K_{0}t$$

K_0_—coefficient of drug release rate for zero order

Higuchi model:

The Higuchi model is the most widely used kinetic model for describing drug release from polymer systems [9,69,70]. Originally developed for low solubility drugs in ointments, it has been extended to semi-solid and solid matrices containing small molecule-based drugs ranging from poorly soluble to highly soluble.

In the Higuchi model, $\frac{M_{t}}{M_{\infty }}$ represents drug released at time t for a specific area. Equation (7) describes the release rate from a saturated matrix.

Equation (7): Higuchi model for saturated matrices

(7) $$\frac{M_{t}}{M_{\infty }}=\sqrt{2C_{0}\varepsilon \frac{Dt}{\tau \pi }}$$

$\frac{M_{t}}{M_{\infty }}$—drug release at time t for specific area
C_0_—initial drug content
ε—matrix porosity
D—diffusion coefficient of the drug
τ—capillary tortuosity factor

Equation (8) is a simplified form of Equation (7), which demonstrates a linear relationship between the square root of time and drug release.

Equation (8): simplified Higuchi model

(8) $$\frac{M_{t}}{M_{\infty }}=K_{H}\sqrt{t}$$

K_H_—release constant

Higuchi model includes the following assumptions:

1. The swelling or dissolution of the matrix is negligible.
2. The diffusivity of the drug is constant.
3. The perfect sink conditions in the release environment.

## 3. Results and Discussion

**Figure 1:**
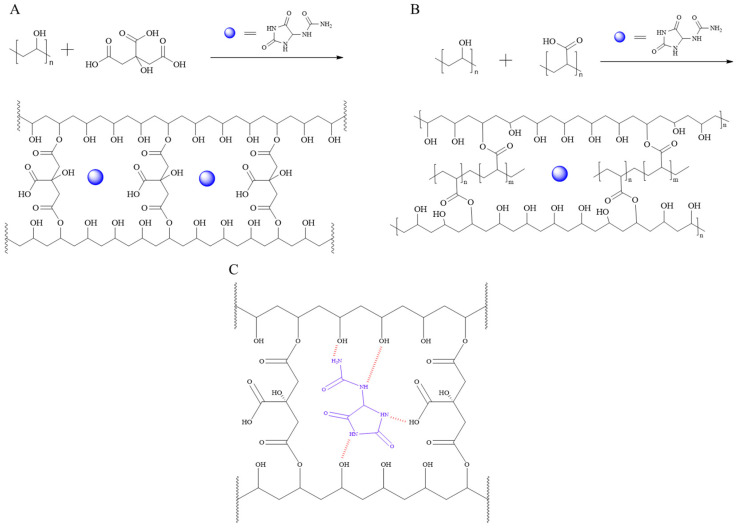
Esterification reactions between PVA and CA (**A**), PVA and PAA (**B**) and electrostatic intermolecular interactions between PVA:CA crosslinked matrix and allantoin (**C**).

Prior fabrication of the coated fabrics, we initially studied processing conditions for the preparation of crosslinked thin films and examined their swelling behavior. Notably, all the materials employed in the preparation of crosslinked films are FDA-approved for human use in food packaging, edible packaging, and delivery systems applied to human skin, posing no concern regarding toxicity, cytotoxicity, or skin reactions within the relevant dosage range [49,53,58]. The crosslinking reactions via esterification between the hydroxyl groups in PVA and carboxylic acid groups in CA and PAA are shown in Figure 1. Formation of chemical and physical crosslinking bonds between PVA and the crosslinkers was accompanied by the formation of hydrogen bonding interactions between allantoin and PVA, PAA, and CA (Figure 1C). Two types of crosslinked films were prepared using 7:3 and 9:1 weight ratios between PVA and CA and PAA crosslinkers as described in the experimental section. The images of the crosslinked films are shown in Figure S2 in the Supporting Information.

### 3.1. Swelling Behavior of the Crosslinked Films

**Figure 2:**
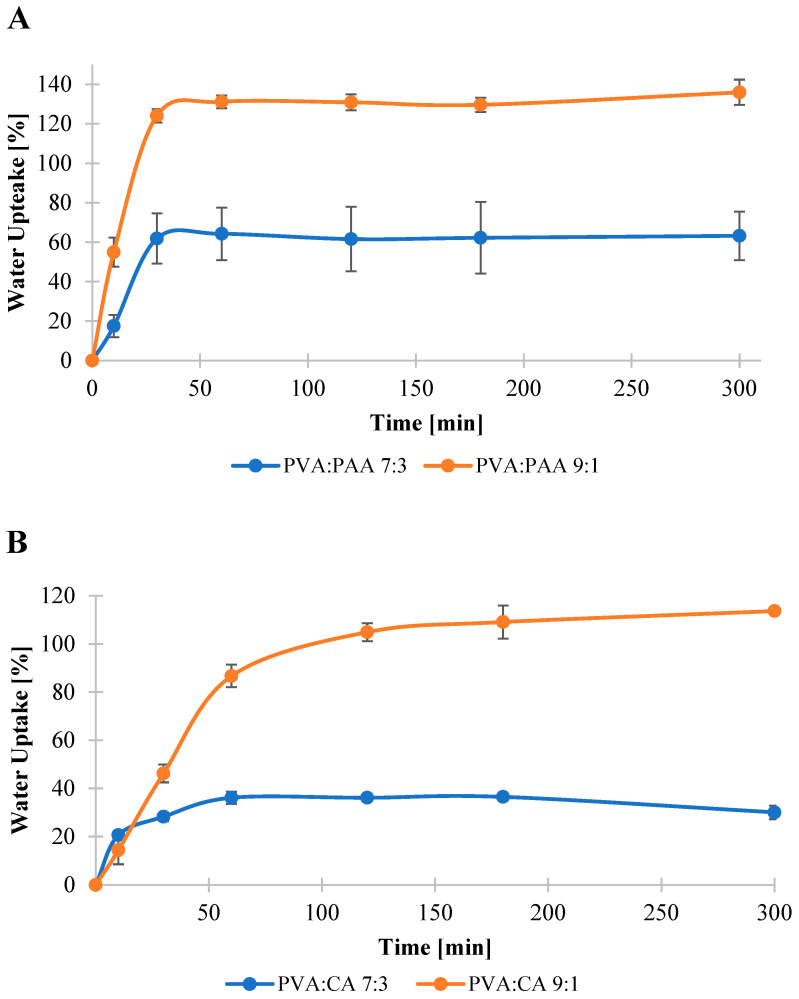
Water uptake of PVA:PAA (**A**) and PVA:CA (**B**) films.

Previous studies on polymer–drug reservoir devices highlighted the critical influence of swelling behavior on the water uptake and dissolution of the entrapped drug molecules in the release media [26,71]. Hence, swelling behavior of the crosslinked films was examined to evaluate their potential to serve as a matrix for the controlled release of allantoin from the coated fabrics. Swelling profiles of the crosslinked films are shown in Figure 2. The results revealed that PVA:PAA crosslinked films exhibited higher water uptake and faster swelling rate in comparison with PVA:CA films. Specifically, the water uptake reached its maximum capacity of 130% after 60 min for PVA:PAA 9:1 and 62% after 30 min for PVA:PAA 7:3 films. In contrast, the water uptake of PVA:CA crosslinked films reached its saturation at 110% for the 9:1 system after 180 min and 36% for the 7:3 system after 60 min.

### 3.2. Gel Content Measurements

The gel content of a crosslinked polymer serves as an indicator of the extent to which polymer chains are covalently/physically linked, forming a compact polymer network. This parameter influences both the degree of water uptake and the ability of a polymer matrix to retain water-soluble active molecules, preventing their migration out of the reservoir system upon exposure to moisture [69,72]. Generally, a higher degree of crosslinking makes it more difficult for water to penetrate the polymer matrix and dissolve the active ingredient. Consistent with the swelling experiments, variations in the gel content of the crosslinked coatings were observed, demonstrating a clear dependence on the crosslinker type and amount (Table 1). The gel content of the PVA:CA 9:1 system was 47%, while it was increased to 83% in the case of PVA:CA 7:3. This difference can be attributed to the higher amount of the CA crosslinker in PVA:PAA 7:3 films and the increased number of the carboxylic acid groups available for the reaction with the hydroxyl groups in PVA. In contrast, the PVA:PAA 9:1 film was completely dissolved in water, exhibiting 0% gel content. Increasing PVA:PAA weight ratio to 7:3 resulted in a gel content of 81%, indicating the formation of covalent and hydrogen bonding between PVA and PAA chains due to the larger number of carboxylic acid moieties in PAA available for the interaction with the hydroxyl groups in PVA.

### 3.3. Termal Analysis

Samples of pre-cured and post-cured coating formulations were tested to study the esterification reaction between PVA and the crosslinker. The changes in the thermal properties of the matrices showed different behaviors for the various polymeric coating formulations (Table 1). For the non-crosslinked PVA:CA, the recorded T_g_ values were 65 °C and 71 °C for the 9:1 and 7:3 systems, respectively. These values are lower compared to the T_g_ of 78 °C measured for the neat, fully hydrolyzed PVA. The lower T_g_ values obtained for the CA-containing PVA films indicate a plasticizing effect of CA on PVA, as also reported in the literature [39,40]. Curing the PVA:CA films at 130 °C resulted in a significant increase in the T_g_ values, to 85 °C and 95 °C for PVA:CA 9:1 and PVA:CA 7:3, respectively. This increase in T_g_ can be attributed to the reduction in the amount of free CA molecules and their plasticizing effect due to the crosslinking reaction, as well as the further restriction of polymer chains movement by the crosslinking bonds due to the formation of covalent ester bonds. In the case of PVA:PAA formulations, the polymers are highly miscible in the non-crosslinked state, resulting in a single T_g_ of 98 °C observed for both 9:1 and 7:3 systems, while neat PAA has T_g_ of 178 °C. However, in the cured films, there was no apparent change in the T_g_ values, indicating that the curing process did not generate significant amount of covalent crosslinking bonds and the films were only slightly chemically crosslinked, containing mostly hydrogen bonding interactions.

### 3.4. Thermo-Mechanical Analysis

Crosslinking density influences key properties of the active agent release mechanism, including water uptake, matrix hydrophilicity, and permeation of small molecules through the matrix. To evaluate the crosslinking density of the coatings, crosslinked PVA:CA and PVA:PAA films were tested using DMA, as described in the experimental section. Equation (3) was employed to calculate the effective M_C_, which represents the averaged molar weight of the chains between crosslinks.

Significant differences in the effective M_C_ values were obtained across all the studied crosslinked formulations, as shown in Table 2. In the case of PVA:CA systems, increasing the amount of CA crosslinker in the coating resulted in a decrease in M_C_ values, from 1045 g/mol to 494 g/mol for PVA:CA 9:1 and 7:3, respectively. This indicates a reduction in the distance between crosslinking points. The Mc value for the PVA:PAA 9:1 system was above 1,000,000 g/mol, indicating that intermolecular interactions between PVA and PAA are mostly based on hydrogen bonds rather than covalent bonds between the two polymers. This outcome is also in good agreement with the gel content value of 0% that was obtained for PVA:PAA 9:1 based coating. On the other hand, PVA:PAA 7:3 crosslinked film exhibited Mc value of 6644 g/mol, revealing a slightly more covalently crosslinked polymer network.

### 3.5. FTIR-ATR Analysis of the Crosslinked PVA Films

**Figure 3:**
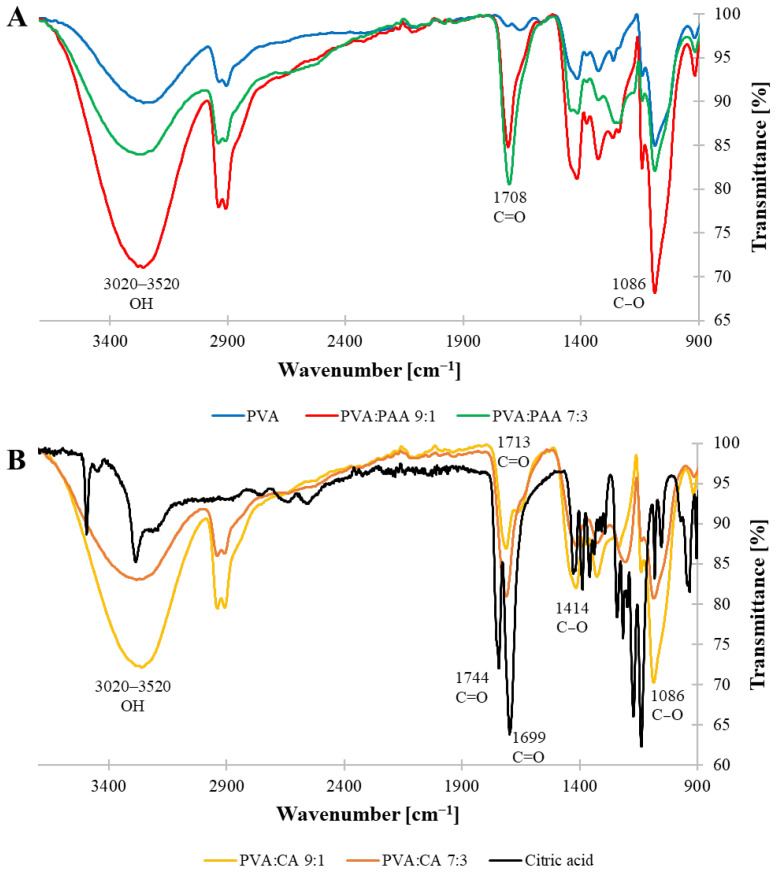
FTIR-ATR spectra of crosslinked films: (**A**) PVA, PVA:PAA 7:3, and PVA:PAA 9:1; (**B**) CA, PVA:CA 7:3, and PVA:CA 9:1.

The FTIR spectra of the crosslinked films were analyzed to gain further insights regarding the formation of the covalent ester bonds in the crosslinked films (Figure 3). The spectra of both PVA:PAA (9:1 and 7:3) films displayed a strong peak at 1708 cm^−1^, corresponding to the C=O moiety of the carboxylic acid groups in PAA (Figure 3A). This signal appeared at a similar wavenumber to the carbonyl group of neat PAA (1702 cm^−1^, Figure S3), indicating that no detectable chemical crosslinking occurred between the carboxylic acid groups and the hydroxyl groups of PVA. These results are in good agreement with the M_C_ values described in the previous section. Furthermore, both spectra displayed a broad signal between 3020 and 3520 cm^−1^, corresponding to the stretching vibration of OH groups, and antisymmetric and symmetric C-H stretch peaks at 2916 and 2848 cm^−1^, respectively, attributed to PVA. Even though the FTIR spectra of both PVA:PAA systems contained similar peaks, a noticeable difference existed in the relative intensity between the carbonyl group of PAA and the stretching vibration of OH groups in both PVA and PAA. While for the 9:1 system the broad band at 3020–3520 cm^−1^ had a stronger intensity relative to the carbonyl signal at 1708 cm^−1^, these signals had the opposite relative intensity in the spectrum of 7:3 system due to the higher concentration of PAA in the film. In the case of CA crosslinker, neat CA showed strong peaks at 1744 and 1699 cm^−1^, attributed to the symmetric vibration of C=O, and at 1419 cm^−1^, corresponding to the asymmetric C-O stretching of the COOH groups (Figure 3B). The signals attributed to neat CA were absent in the spectra of both PVA:CA crosslinked films, which displayed a carbonyl group peak at 1713 cm^−1^ indicating formation of ester linkages in the crosslinked films. Furthermore, the intensity of the carbonyl signal relative to the wide band at 3020–3520 cm^−1^ was stronger for the 7:3 system, reflecting the higher number of covalent crosslinks in this system compared to the 9:1 one.

### 3.6. Fabric Coating and Determination of Crosslinked Coating and Allantoin Contents

**Figure 4:**
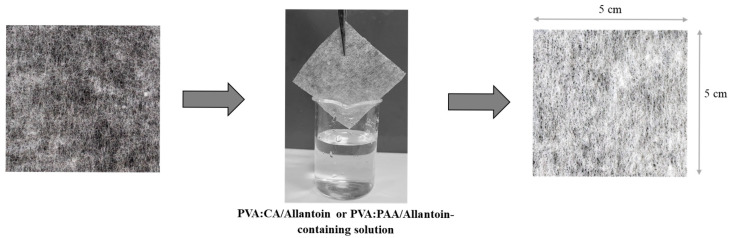
Schematic representation of coating procedure of nonwoven fabrics using aqueous solution containing allantoin and PVA, CA, or AA crosslinkers.

To fabricate coated polyethylene nonwoven fabrics, fabric samples were dip-coated using aqueous solutions of the crosslinking formulations containing allantoin as described in the experimental section (Figure 4).

The thickness of the coatings and the amount of active ingredient within the fabric sample may potentially influence the release profile of the active reagent [26]. To estimate coating accumulation and allantoin content in the coated fabrics, fabric samples (4 × 4 cm^2^) were weighed before and after soaking in the crosslinking solutions, followed by curing. The results are summarized in Table 3. Fabric samples coated with PVA:CA formulations exhibited coating mass of 42 and 39 mg for the 9:1 and 7:3 systems, respectively. On the other hand, coating mass values were almost double for PVA:PAA formulations were 88 and 80 mg for 9:1 and 7:3 systems, respectively. Coating thickness was approximately 250 µm for the PVA:CA and 330 µm for the PVA:PAA coated fabrics. The higher coating mass and thickness obtained for the PVA:PAA coated fabrics can be attributed to the higher viscosities of the coating formulations [73]. While the viscosities of the PVA:PAA-based formulations ranged from 220 to 250 cP, PVA:CA formulations had significantly lower viscosity values of 8–10 cP, resulting in the formation of thinner crosslinked coatings.

In addition, we found a strong correlation between the thickness of the crosslinked coating and the amount of allantoin incorporated into the fabrics, which was estimated via the accumulative amount of allantoin obtained from release experiments, as described in the experimental section. The coating thickness measured for the PVA:PAA and PVA:CA coated fabrics was 330 µm and 250 µm, respectively. The higher coating thickness obtained for the PVA:PAA based coatings allowed incorporation of higher amount of allantoin in comparison with the PVA-CAA ones as shown in Table 3. In addition, both PVA:PAA and PVA:CA systems exhibited higher amount of allantoin in the coatings prepared using a 9:1 ratio between PVA and the crosslinker relatively to the 7:3 based systems. In fact, when a 9:1 ratio between PVA and CA was used, the allantoin mass was 1.8 mg, and it was decreased to 1.2 mg for the 7:3 system. PVA:PAA coated fabrics showed a similar trend and contained 4.2 mg and 2.6 mg of allantoin in the 9:1 and 7:3 based coatings, respectively. These behaviors can be attributed to the fact that the 9:1 systems have lower crosslinking density than the 7:3 ones, providing more free volume for the incorporation for allantoin.

### 3.7. Water Contact Angle Measurements

To examine surface wettability of the coated fabrics, water contact angle measurements were carried out and the images of the fabric samples obtained during the test are shown in Figure S4. The unmodified polyethylene fabric exhibited hydrophobic characteristics, with a water contact angle of 133 ± 2°. A complete wetting was observed for the fabrics coated with all types of the crosslinked formulations before curing, due to the hydrophilicity of PVA and the crosslinker molecules. Following the curing process, which promoted PVA crosslinking and the consumption of free hydroxyl and carboxylic acid groups, the water contact angles of PVA:CA 9:1 were increased to 79 ± 5°, with an additional increase to 111 ± 7° for the more crosslinked PVA:CA 7:3 coated fabric. In contrast, the crosslinking process in the PVA:PAA-based coating did not result in an increase in hydrophobicity, and the surface of the coated fabrics remained hydrophilic. The hydrophilic nature of the PAA-based coated fabrics can be attributed to almost negligible extent of the esterification reaction between PVA and PAA, leading to the retention of the carboxylic acid and hydroxyl groups on the surface of the cured fabrics. This surface wettability behavior combined with the results obtained from the FTIR-ATR and Mc measurements, supports the conclusion that PAA-based crosslinked systems contained mainly hydrogen bonding interactions between PVA and PAA.

### 3.8. SEM Analysis

**Figure 5:**
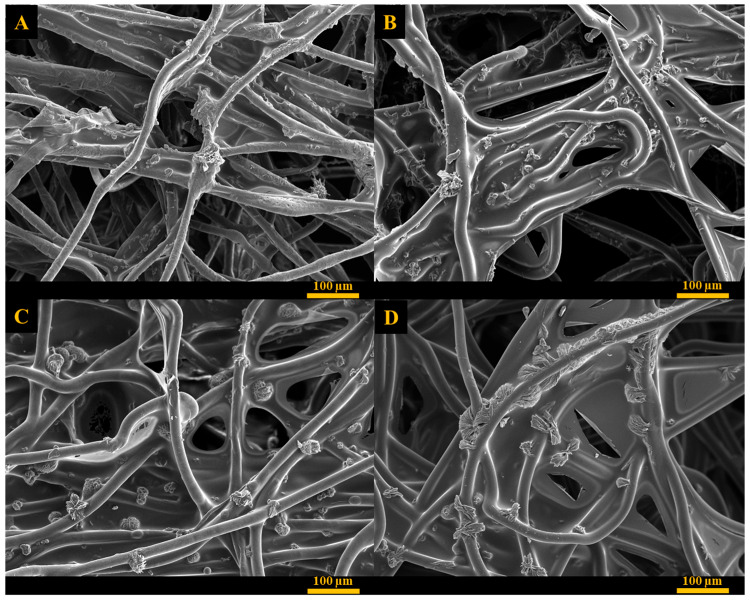
SEM images of (**A**) PVA:CA 9:1, (**B**) PVA:CA 7:3, (**C**) PVA:PAA 9:1, (**D**) PVA:PAA 7:3.

The surface morphology of the coated fabrics was studied using SEM and the images of the unmodified and coated fabrics containing allantoin are shown in Figure 5 and Figure S5. The surface of the uncoated fabric displayed needle-shape allantoin crystals with average dimensions of 39 µm in length and 10 µm in width. Allantoin crystals were also observed on the surface of all coated fabrics, indicating that allantoin concentration within the polymer network was above its saturation point. According to the previous studies, this is one of the essential factors for maintaining prolonged and constant release rate of the active material [69,72]. The coatings appeared as thin discontinuous films incorporating allantoin crystals that were randomly distributed in the coating and appeared on the surface and between polyethylene fibers. Allantoin crystals formed aggregates with irregular shape and average size of 14 µm in length and 35 µm in width for PVA:CA 7:3, and 15 µm in length and 17 µm in width for the PVA:CA 9:1 coated fabrics. Fabrics coated using PVA:PAA formulations exhibited a significantly more extensive and continuous polymer film covering polyethylene fibers and larger amount of allantoin in comparison with PVA:CA based coatings. In this case, allantoin crystals created spherulite-shaped aggregates with an average diameter of 32 µm and 37 µm for 9:1 and 7:3 based coatings, respectively.

### 3.9. Allantoin Release

**Figure 6:**
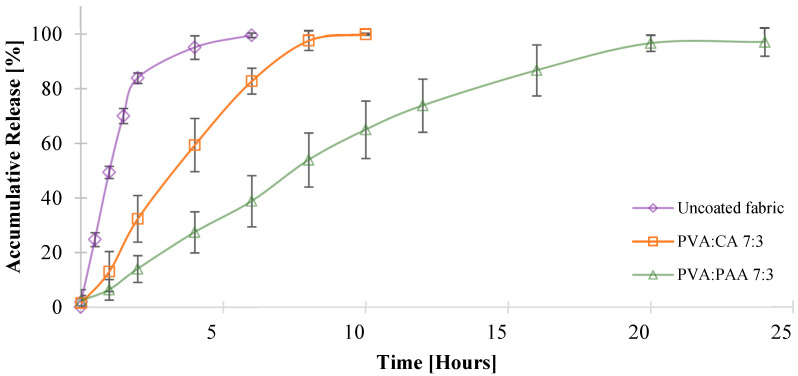
In vitro evaluation of allantoin release using PVA:PAA 7:3 and PVA:CA 7:3 coated fabrics.

The release profiles of allantoin from the coated fabrics were obtained using a vertical Franz diffusion cell under sink conditions, as described in the experimental section. Fabrics treated with PVA:PAA 9:1 and PVA:CA 9:1 formulations exhibited a burst release of allantoin, with over 60% of the allantoin released within the first hour, followed by a 90% cumulative release after 10 and 6 h, respectively (Figure S6). This burst release behavior can be attributed to the low crosslinking density of the coatings containing a 9:1 ratio between PVA and crosslinker, resulting in enhanced water permeability. In contrast, fabrics coated using PVA:CA 7:3 and PVA:PAA 7:3 formulations demonstrated controlled release of allantoin for 8 and 20 h, respectively (Figure 6).

PVA:PAA 7:3 and PVA:CA 7:3 coatings exhibited a much higher gel content and decreased water uptake rates compared to the 9:1 systems. The difference in the release profiles of PVA:CA 7:3 and PVA:PAA 7:3 can be attributed to the different viscosities of the coating formulations, that influenced the coating thickness and the amount of allantoin incorporated into the fabric. While fabrics coated with the PVA:CA 7:3 formulation had a 250 ± 40 µm coating thickness and a total amount of 1.2 mg of allantoin, fabrics coated with PVA:PAA 7:3 formulation had a 330 ± 50 µm coating thickness and released a total amount of 2.6 mg of allantoin. These results are consistent with previously reported studies, which described a higher amount of active agent incorporation and a more prolonged release rate for thicker crosslinked polymer matrices compared to thinner ones [71,74].

Finally, we examined the kinetics of allantoin release from PVA:PAA 7:3 and PVA:CA 7:3 coated fabrics using four mathematical models: Zero-order, First-order, Higuchi, and Ritger–Peppas, as presented in Equations (9)–(12). According to the literature, these kinetic models are valid only for up to 60% of the drugs released under sink conditions in the release environment [67,69]. Therefore, PVA:CA 9:1 and PVA:PAA 9:1 were excluded from this analysis due to the burst release of allantoin observed for these systems.

(9) $$\mathrm{Ritger}–\mathrm{Peppas}\;\frac{M_{t}}{M_{\infty }}=Kt_{n}$$

(10) $$\mathrm{Zero}\;\mathrm{order}\;\frac{M_{t}}{M_{\infty }}=K_{0}t$$

(11) $$\mathrm{Higuchi}\;\frac{M_{t}}{M_{\infty }}=K_{H}\sqrt{t}$$

(12) $$\mathrm{First}\;\mathrm{order}\;\ln\left(1-\frac{M_{t}}{M_{\infty }}\right)=-K_{1}t$$

where M_t_/M_∞_ represents the fractional drug release at time t; K, K_0_, K_H_, and K_1_ are the release constants, dependent on the polymer matrix; t is the release time; and n is the diffusional exponent.

A summary of the coefficient of determination (R^2^) values and other parameters related to the mathematical models is presented in Table 4. The results revealed that the release profiles of both PVA:PAA 7:3 and PVA:CA 7:3 systems were in good agreement with the Zero-order model, as reflected by R^2^ values of 0.9931 and 0.9963 for the PVA:CA 7:3 and PVA-PAA 7:3 systems, respectively (Figure S7).

These results can be explained by examining the key parameters that influence the release profile from crosslinked thin films and hydrogels: the water uptake/swelling behavior of the crosslinked system and the solubility of the released active agent within the crosslinked matrix [75,76]. PVA:CA 7:3 and PVA:PAA 7:3 coatings exhibited water uptake of 36% within 60 min and 62% within 30 min, respectively, until reaching the maximum capacity and remaining constant throughout the entire release period. These results show better compliance with type II, non-Fickian solute transport from the crosslinked polymers, which comply with their ability to swell rapidly and undergo chain relaxation during the active agent release process, alongside other factors such as saturation of the active molecule in the polymer system [69,77]. In addition, one of the requirements of Zero-order release kinetics is that the drug concentration in the matrix must be above the saturation point, and the reservoir must exceed the saturation point within the drug release time range, while the active agent amount in the release medium remains below saturation [75,78]. The allantoin concentration in the coated fabrics likely exceeded the saturation point for both PVA:PAA 7:3 and PVA:CA 7:3 matrices, as indicated by the crystalline allantoin particles observed in the SEM images of the crosslinked fabrics (Figure 5).

## 4. Conclusions

Crosslinked PVA-based coating formulations for PE nonwoven fabrics were successfully developed for the incorporation and controlled release of hydrophilic agent, allantoin, upon exposure to water. The allantoin release profiles from the coated fabrics were strongly dependent on the type of crosslinker and on the composition of the crosslinked coating, both of which influenced crosslinking density and water uptake. When the PVA:PAA weight ratio was 9:1, the coating exhibited 0% gel content and an M_C_ value above 1,000,000, indicating mainly hydrogen bonding interactions between the polymers. Although the PVA:CA 9:1 coating had a higher crosslinking density than the PVA:PAA 9:1 one, a rapid water uptake was observed for both coatings, resulting in the burst release of allantoin. On the other hand, effectively crosslinked polymer networks with approximately 80% gel content and limited water uptake were achieved for the PVA:PAA 7:3 and PVA:CA 7:3 systems. The release profiles from the coated fabrics prepared using PVA:PAA 7:3 and PVA:CA 7:3 coating formulations complied with the Zero-order model, facilitating continuous allantoin release for up to 8 and 20 h for PVA:CA 7:3 and PVA:PAA 7:3 coatings, respectively. The results of this study indicate that PVA:CA and PVA:PAA formulations can serve as a general strategy for reducing the aqueous solubility of hydrophilic molecules encapsulated within crosslinked polymer coating through intermolecular bonding interactions. The efficient and practically simple coating method of nonwoven fabrics and the use of biocompatible materials in the coating formulations make this system promising for bio-medical and pharma applications such as smart wound dressings, face masks and bandages, that require controlled release of hydrophilic therapeutic and cosmetic compounds to skin.

## Figures and Tables

**Table 1:** Gel content and T_g_ values for the crosslinked films before and after curing.

Coating Formulation	Gel Content [%]	T_g_ [°C] Before Curing	T_g_ [°C] After Curing
PVA:CA 9:1	47 ± 4	65	85
PVA:CA 7:3	83 ± 3	71	95
PVA:PAA 9:1	0	98	94
PVA:PAA 7:3	81 ± 2	98	99

**Table 2:** Storage modulus and M_C_ values of the crosslinked PVA:CA and PVA:PAA films.

Crosslinked Films	E′ [Mpa]	$\rho_{polymer}$ [g/mL]	M_C_ [g/mol]
PVA:CA 9:1	15	1.33 ± 0.01	1045
PVA:CA 7:3	29	1.34 ± 0.02	494
PVA:PAA 9:1	0.01	1.31 ± 0.01	>1,000,000
PVA:PAA 7:3	2	1.38 ± 0.03	6644

**Table 3:** Summary of parameters of the crosslinked PVA:CA and PAA:PAA coatings.

Coating Formulation	Coating Mass [mg]	Coating Thickness [µm]	Coating Viscosity [cP]	Allantoin Mass [mg]
PVA:CA 9:1	42 ± 6	250 ± 10	10	1.8 ± 0.3
PVA:CA 7:3	39 ± 8	250 ± 40	8	1.2 ± 0.3
PVA:PAA 9:1	88 ± 3	330 ± 0	250	4.2 ± 0.3
PVA:PAA 7:3	80 ± 10	330 ± 50	220	2.6 ± 0.2

**Table 4:** Mathematical modeling of allantoin release profile.

Coated Fabric	Zero-Order	First-Order	Higuchi	Ritger–Peppas		
	R^2^	R^2^	R^2^	R^2^	K	n
PVA:CA 7:3	0.9931	0.9015	0.8748	0.9804	1.1690	0.7080
PVA:PAA 7:3	0.9963	0.9352	0.8855	0.9872	0.4717	0.7581

## Data Availability

The original contributions presented in this study are included in the article; further inquiries can be directed to the corresponding author.

## Supplementary Materials

The following supporting information can be downloaded at: https://www.mdpi.com/article/10.3390/jfb16060216/s1. Figure S1: Allantoin Release Assessment—calibration curve; Figure S2: Images of the crosslinked thin films for different coating formulations; Figure S3: FTIR-ATR spectra of neat poly acrylic acid; Figure S4: (A) polyethylene fabric neat; (B) polyethylene fabric coated with PVA and cured in 130 °C for 2-h; (C) polyethylene fabric coated in PVA:CA 9:1; and (D) polyethylene fabric coated in PVA:CA 7:3; Figure S5: SEM image of polyethylene fabric (A) neat and (B) containing 15 wt% of allantoin; Figure S6: In-vitro drug release evaluation from PVA:PAA 9:1 and PVA:CA 9:1 formulation; Figure S7: Comparison between (A) PVA:CA 7:3 and (B) PVA:PAA 7:3 to zero-order release kinetics.

## References

[1] Gupta B.S. (1998). Medical Textile Structures: An Overview. Med. Device Diagn. Ind..

[2] Atanasova D., Staneva D., Grabchev I. (2021). Textile Materials Modified with Stimuli-Responsive Drug Carrier for Skin Topical and Transdermal Delivery. Materials.

[3] Abhishesh K.M., Deepa D., Vikas, Vishnu P., Madaswamy S.M., Navneet S., Bhupendra S.B. (2023). Drug-releasing textile materials: Current developments and future perspectives. Fiber and Textile Engineering in Drug Delivery Systems.

[4] Qin Y., Qin Y. (2016). An overview of medical textile products. Medical Textile Materials.

[5] Pinho E., Soares G. (2018). Functionalization of cotton cellulose for improved wound healing. J. Mater. Chem. B.

[6] Martí M., Alonso C., Martínez V., Lis M., de la Maza A., Parra J.L., Coderch L. (2013). Cosmetotextiles with Gallic Acid: Skin Reservoir Effect. J. Drug Deliv..

[7] Lis M.J., Martí M., Coderch L., Alonso C., Bezerra F.M., Immich A.P., Tornero J.A. (2019). Biofunctional Textiles. Advances in Textile Engineering.

[8] Lis Arias M.J., Coderch L., Martí M., Alonso C., Carmona O.G., Carmona C.G., Maesta F. (2018). Vehiculation of Active Principles as a Way to Create Smart and Biofunctional Textiles. Materials.

[9] Rostamitabar M., Abdelgawad A.M., Jockenhoevel S., Ghazanfari S. (2021). Drug-Eluting Medical Textiles: From Fiber Production and Textile Fabrication to Drug Loading and Delivery. Macromol. Biosci..

[10] Amjadi M., Sheykhansari S., Nelson B.J., Sitti M. (2018). Recent Advances in Wearable Transdermal Delivery Systems. Adv. Mater..

[11] Massella D., Argenziano M., Ferri A., Guan J., Giraud S., Cavalli R., Barresi A.A., Salaün F. (2019). Bio-Functional Textiles: Combining Pharmaceutical Nanocarriers with Fibrous Materials for Innovative Dermatological Therapies. Pharmaceutics.

[12] ten Breteler M.R., Nierstrasz V.A., Warmoeskerken M.M.C.G. (2002). Textile Slow-Release Systems With Medical Applications. AUTEX Res. J..

[13] Shergill B., Zokaie S., Carr A.J. (2014). Non-adherence to topical treatments for actinic keratosis. Patient Prefer. Adherence.

[14] Tan X., Feldman S.R., Chang J., Balkrishnan R. (2012). Topical drug delivery systems in dermatology: A review of patient adherence issues. Expert Opin. Drug Deliv..

[15] Devaux S., Castela A., Archier E., Gallini A., Joly P., Misery L., Aractingi S., Aubin F., Bachelez H., Cribier B. (2012). Adherence to topical treatment in psoriasis: A systematic literature review. J. Eur. Acad. Dermatol. Venereol..

[16] Yélamos O., Ros S., Puig L. (2015). Improving patient outcomes in psoriasis: Strategies to ensure treatment adherence. Psoriasis Targets Ther..

[17] Frederiksen K., Guy R.H., Petersson K. (2015). Formulation considerations in the design of topical, polymeric film-forming systems for sustained drug delivery to the skin. Eur. J. Pharm. Biopharm..

[18] Petrusic S., Koncar V., Koncar V. (2016). Controlled release of active agents from microcapsules embedded in textile structures. Smart Textiles and Their Applications.

[19] Qiao N., Zhang Y., Fang Y., Deng H., Zhang D., Lin H., Chen Y., Yong K.T., Xiong J. (2022). Silk Fabric Decorated with Thermo-Sensitive Hydrogel for Sustained Release of Paracetamol. Macromol. Biosci..

[20] Puoci F., Saturnino C., Trovato V., Iacopetta D., Piperopoulos E., Triolo C., Bonomo M.G., Drommi D., Parisi O.I., Milone C. (2020). Sol-Gel Treatment of Textiles for the Entrapping of an Antioxidant/Anti-Inflammatory Molecule: Functional Coating Morphological Characterization and Drug Release Evaluation. Appl. Sci..

[21] Hashemikia S., Hemmatinejad N., Ahmadi E., Montazer M. (2016). A novel cotton fabric with anti-bacterial and drug delivery properties using SBA-15-NH_2_/polysiloxane hybrid containing tetracycline. Mater. Sci. Eng. C.

[22] Gu P., Li B., Wu B., Wang J., Müller-Buschbaum P., Zhong Q. (2020). Controlled Hydration, Transition, and Drug Release Realized by Adjusting Layer Thickness in Alginate-Ca^2+^/poly(N-isopropylacrylamide) Interpenetrating Polymeric Network Hydrogels on Cotton Fabrics. ACS Biomater. Sci. Eng..

[23] Nikdel M., Rajabinejad H., Yaghoubi H., Mikaeiliagah E., Cella M.A., Sadeghianmaryan A., Ahmadi A. (2021). Fabrication of Cellulosic Nonwoven Material Coated with Polyvinyl Alcohol and Zinc Oxide/Mesoporous Silica Nanoparticles for Wound Dressing Purposes with Cephalexin Delivery. ECS J. Solid State Sci. Technol..

[24] Ehsani A., Asefnejad A., Sadeghianmaryan A., Rajabinejad H., Chen X. (2021). Fabrication of Wound Dressing Cotton Nano-Composite Coated with Tragacanth/Polyvinyl Alcohol: Characterization and In Vitro Studies. ECS J. Solid State Sci. Technol..

[25] Hartman C., Popowski Y., Raichman D., Amir E. (2020). Biodegradable polymer coating for controlled release of hydrophobic functional molecules from cotton fabrics. J. Coatings Technol. Res..

[26] Maderuelo C., Zarzuelo A., Lanao J.M. (2011). Critical factors in the release of drugs from sustained release hydrophilic matrices. J. Control. Release.

[27] Mostafalu P., Kiaee G., Giatsidis G., Khalilpour A., Nabavinia M., Dokmeci M.R., Sonkusale S., Orgill D.P., Tamayol A., Khademhosseini A. (2017). A Textile Dressing for Temporal and Dosage Controlled Drug Delivery. Adv. Funct. Mater..

[28] Montaser A.S., Rehan M., El-Senousy W.M., Zaghloul S. (2020). Designing strategy for coating cotton gauze fabrics and its application in wound healing. Carbohydr. Polym..

[29] Andleeb A., Dikici S., Waris T.S., Bashir M.M., Akhter S., Chaudhry A.A., MacNeil S., Yar M. (2020). Developing affordable and accessible pro-angiogenic wound dressings; incorporation of 2 deoxy D-ribose (2dDR) into cotton fibres and wax-coated cotton fibres. J. Tissue Eng. Regen. Med..

[30] Goudarzi Z.M., Soleimani M., Ghasemi-Mobarakeh L., Sajkiewicz P., Sharifianjazi F., Esmaeilkhanian A., Khaksar S. (2022). Control of drug release from cotton fabric by nanofibrous mat. Int. J. Biol. Macromol..

[31] Fu Y.C., Ferng L.H.A., Huang P.Y. (2006). Quantitative analysis of allantoin and allantoic acid in yam tuber, mucilage, skin and bulbil of the Dioscorea species. Food Chem..

[32] Haghi G., Arshi R., Safaei A. (2008). Improved High-Performance Liquid Chromatography (HPLC) Method for Qualitative and Quantitative Analysis of Allantoin in *Zea mays*. J. Agric. Food Chem..

[33] Sugarman J.L. (2008). The Epidermal Barrier in Atopic Dermatitis. Semin. Cutan. Med. Surg..

[34] Gao X.H., Zhang L., Wei H., Chen H.D. (2008). Efficacy and safety of innovative cosmeceuticals. Clin. Dermatol..

[35] Gordon M.L. (1998). The Role of Clobetasol Propionate Emollient 0.05% in the Treatment of Patients with Dry, Scaly, Corticosteroid-Responsive Dermatoses. Clin. Ther..

[36] Gagari E., Kabani S. (1995). Adverse effects of mouthwash use: A review. Oral Surg. Oral Med. Oral Pathol. Oral Radiol. Endodontology.

[37] Dyer D.L., Gerenratch K.B., Wadhams P.S. (1998). Testing a New Alcohol-Free Hand Sanitizer to Combat Infection. AORN J..

[38] Karagoz H., Yuksel F., Ulkur E., Evinc R. (2009). Comparison of efficacy of silicone gel, silicone gel sheeting, and topical onion extract including heparin and allantoin for the treatment of postburn hypertrophic scars. Burns.

[39] Thomas L.V., Arun U., Remya S., Nair P.D. (2009). A biodegradable and biocompatible PVA-citric acid polyester with potential applications as matrix for vascular tissue engineering. J. Mater. Sci. Mater. Med..

[40] Birck C., Degoutin S., Tabary N., Miri V., Bacquet M. (2014). New crosslinked cast films based on poly(vinyl alcohol): Preparation and physico-chemical properties. Express Polym. Lett..

[41] Zhang K., Liu Y., Shi X., Zhang R., He Y., Zhang H., Wang W. (2023). Application of polyvinyl alcohol/chitosan copolymer hydrogels in biomedicine: A review. Int. J. Biol. Macromol..

[42] Xiao M., Tan M., Peng C., Jiang F., Wu K., Liu N., Li D., Yao X. (2024). Soft and flexible polyvinyl alcohol/pullulan aerogels with fast and high water absorption capacity for facial mask substrates. Int. J. Biol. Macromol..

[43] Thanh N.Q., Mai D.H., Le T.P.A., Do N.H.N., Le P.K. (2025). Novel chitosan/polyvinyl alcohol gel encapsulating ethanolic Centella asiatica extract for cosmeceutical applications. Polym. Bull..

[44] Mitura S., Sionkowska A., Jaiswal A. (2020). Biopolymers for hydrogels in cosmetics: Review. J. Mater. Sci. Mater. Med..

[45] Bai Z., Wang T., Zheng X., Huang Y., Chen Y., Dan W. (2021). High strength and bioactivity polyvinyl alcohol/collagen composite hydrogel with tannic acid as cross-linker. Polym. Eng. Sci..

[46] Asthana N., Pal K., Aljabali A.A.A., Tambuwala M.M., de Souza F.G., Pandey K. (2021). Polyvinyl alcohol (PVA) mixed green–clay and aloe vera based polymeric membrane optimization: Peel-off mask formulation for skin care cosmeceuticals in green nanotechnology. J. Mol. Struct..

[47] Rivera-Hernández G., Antunes-Ricardo M., Martínez-Morales P., Sánchez M.L. (2021). Polyvinyl alcohol based-drug delivery systems for cancer treatment. Int. J. Pharm..

[48] Nigro L., Magni S., Ortenzi M.A., Gazzotti S., Della Torre C., Binelli A. (2022). Are “liquid plastics” a new environmental threat? The case of polyvinyl alcohol. Aquat. Toxicol..

[49] Castro J.M., Montalbán M.G., Martínez-Pérez N., Domene-López D., Pérez J.M., Arrabal-campos F.M., Fernández I., Martín-Gullón I., García-Quesada J.C. (2023). Thermoplastic starch/polyvinyl alcohol blends modification by citric acid–glycerol polyesters. Int. J. Biol. Macromol..

[50] Sharma P., Kumar Agrawal P., Singh V.K., Chauhan S., Bhaskar J. (2023). A Comprehensive Review on Properties of Polyvinyl Alcohol (PVA) Crosslinked with Carboxylic Acid. J. Mater. Environ. Sci..

[51] Saraiva M.M., Campelo M.D.S., Câmara Neto J.F., Lima A.B.N., Silva G.D.A., Dias A.T.D.F.F., Ribeiro M.E.N.P. (2023). Alginate/polyvinyl alcohol films for wound healing: Advantages and challenges. J. Biomed. Mater. Res. Part B Appl. Biomater..

[52] DeMerlis C.C., Schoneker D.R. (2003). Review of the oral toxicity of polyvinyl alcohol (PVA). Food Chem. Toxicol..

[53] Shi R., Bi J., Zhang Z., Zhu A., Chen D., Zhou X., Zhang L., Tian W. (2008). The effect of citric acid on the structural properties and cytotoxicity of the polyvinyl alcohol/starch films when molding at high temperature. Carbohydr. Polym..

[54] Jaeger T., Rothmaier M., Zander H., Ring J., Gutermuth J., Anliker M.D. (2015). Acid-coated Textiles (pH 5.5–6.5)—A New Therapeutic Strategy for Atopic Eczema?. Acta. Derm. Venereol..

[55] Vukušić S.B., Grgac S.F., Budimir A., Kalenić S. (2011). Cotton textiles modified with citric acid as efficient anti-bacterial agent for prevention of nosocomial infections. Croat. Med. J..

[56] Chung Y.S., Lee K.K., Kim J.W. (1998). Durable Press and Antimicrobial Finishing of Cotton Fabrics with a Citric Acid and Chitosan Treatment. Text. Res. J..

[57] Książek E. (2024). Citric Acid: Properties, Microbial Production, and Applications in Industries. Molecules.

[58] Wang L., Duan L., Liu G., Sun J., Shahbazi M.A., Kundu S.C., Reis R.L., Xiao B., Yang X. (2023). Bioinspired Polyacrylic Acid-Based Dressing: Wet Adhesive, Self-Healing, and Multi-Biofunctional Coacervate Hydrogel Accelerates Wound Healing. Adv. Sci..

[59] Zahran M.A., Abd El-Mawgood W.A., Basuni M.M. (2016). Poly Acrylic Acid: Synthesis, aqueous Properties and their Applications as scale Inhibitor. Kautsch. Gummi Kunststoffe.

[60] Barsbay M., Güven O. (2013). RAFT mediated grafting of poly(acrylic acid) (PAA) from polyethylene/polypropylene (PE/PP) nonwoven fabric via preirradiation. Polymer.

[61] Arkaban H., Barani M., Akbarizadeh M.R., Pal Singh Chauhan N., Jadoun S., Dehghani Soltani M., Zarrintaj P. (2022). Polyacrylic Acid Nanoplatforms: Antimicrobial, Tissue Engineering, and Cancer Theranostic Applications. Polymers.

[62] Barszczewska-Rybarek I.M., Korytkowska-Wałach A., Kurcok M., Chladek G., Kasperski J. (2017). DMA analysis of the structure of crosslinked poly(methyl methacrylate)s. Acta. Bioeng. Biomech..

[63] Krumova M., López D., Benavente R., Mijangos C., Pereña J.M. (2000). Effect of crosslinking on the mechanical and thermal properties of poly(vinyl alcohol). Polymer.

[64] Leistner C., Hartmann S., Wittrock J., Bode K. (2018). Shrinkage behavior of Araldite epoxy resin using Archimedes’ principle. Polym. Test..

[65] Fathollahipour S., Koosha M., Tavakoli J., Maziarfar S., Mehrabadi J.F. (2020). Erythromycin Releasing PVA/sucrose and PVA/honey Hydrogels as Wound Dressings with Antibacterial Activity and Enhanced Bio-adhesion. Iran. J. Pharm. Res..

[66] Zahra F.T., Quick Q., Mu R. (2023). Electrospun PVA Fibers for Drug Delivery: A Review. Polymers.

[67] Ritger P.L., Peppas N.A. (1987). A simple equation for description of solute release I. Fickian and non-fickian release from non-swellable devices in the form of slabs, spheres, cylinders or discs. J. Control. Release.

[68] Kusjuriansah K., Rodhiyah M., Syifa N.A., Luthfianti H.R., Waresindo W.X., Hapidin D.A., Suciati T., Edikresnha D., Khairurrijal K. (2024). Composite Hydrogel of Poly(vinyl alcohol) Loaded by Citrus hystrix Leaf Extract, Chitosan, and Sodium Alginate with In Vitro Antibacterial and Release Test. ACS Omega.

[69] Bruschi M.L. (2015). Mathematical models of drug release. Strategies to Modify the Drug Release from Pharmaceutical Systems.

[70] Martí M., Martínez V., Lis M.J., Coderch L. (2021). Mathematical models for drug delivery from textile. J. Ind. Text..

[71] Joshi H.A., Doiphode O.A., Jadhav R.V., Patil R.N. (2016). Hydrogel Drug Delivery System. Pharmacophore.

[72] Dash S., Murthy P.N., Nath L., Chowdhury P. (2010). Kinetic modeling on drug release from controlled drug delivery systems. Acta Pol. Pharm. Drug Res..

[73] Brinker C.J., Schneller T., Waser R., Kosec M., Payne D. (2013). Dip Coating. Chemical Solution Deposition of Functional Oxide Thin Films.

[74] Bruschi M.L. (2015). Main mechanisms to control the drug release. Strategies to Modify the Drug Release from Pharmaceutical Systems.

[75] Lavrentev F.V., Shilovskikh V.V., Alabusheva V.S., Yurova V.Y., Nikitina A.A., Ulasevich S.A., Skorb E.V. (2023). Diffusion-Limited Processes in Hydrogels with Chosen Applications from Drug Delivery to Electronic Components. Molecules.

[76] Thang N.H., Chien T.B., Cuong D.X. (2023). Polymer-Based Hydrogels Applied in Drug Delivery: An Overview. Gels.

[77] Serra L., Doménech J., Peppas N.A. (2006). Drug transport mechanisms and release kinetics from molecularly designed poly(acrylic acid-g-ethylene glycol) hydrogels. Biomaterials.

[78] Adepu S., Ramakrishna S. (2021). Controlled Drug Delivery Systems: Current Status and Future Directions. Molecules.

