# Supplementary material for: Controlled Release of Hydrophilic Active Agent from Textile Using Crosslinked Polyvinyl Alcohol Coatings
Source: J Funct Biomater. 2025 Jun 10;16(6):216. doi: 10.3390/jfb16060216 (PMC12193727; doi:10.3390/jfb16060216)
Supplement: Supplementary file 1 [file jfb-16-00216-s001.zip › jfb-3603997-supplementary.pdf]

Article

Controlled release of hydrophilic active agent from textile using crosslinked polyvinyl alcohol coatings

Limor Mizrahi[1], Rotem Kelman[1], Efrat Shtriker[1], David Meridor[1], Dror Cohen[2], Meital Portugal-Cohen[2] and Elizabeth Amir[1*]

[1] Department of Polymer Materials Engineering, Shenkar College, 5252626 Ramat-Gan, Israel
[2] AHAVA Dead Sea Laboratories LTD, 1 Arava, Airport City 7019900, Israel
* Correspondence: eamir@shenkar.ac.il

Supporting information:

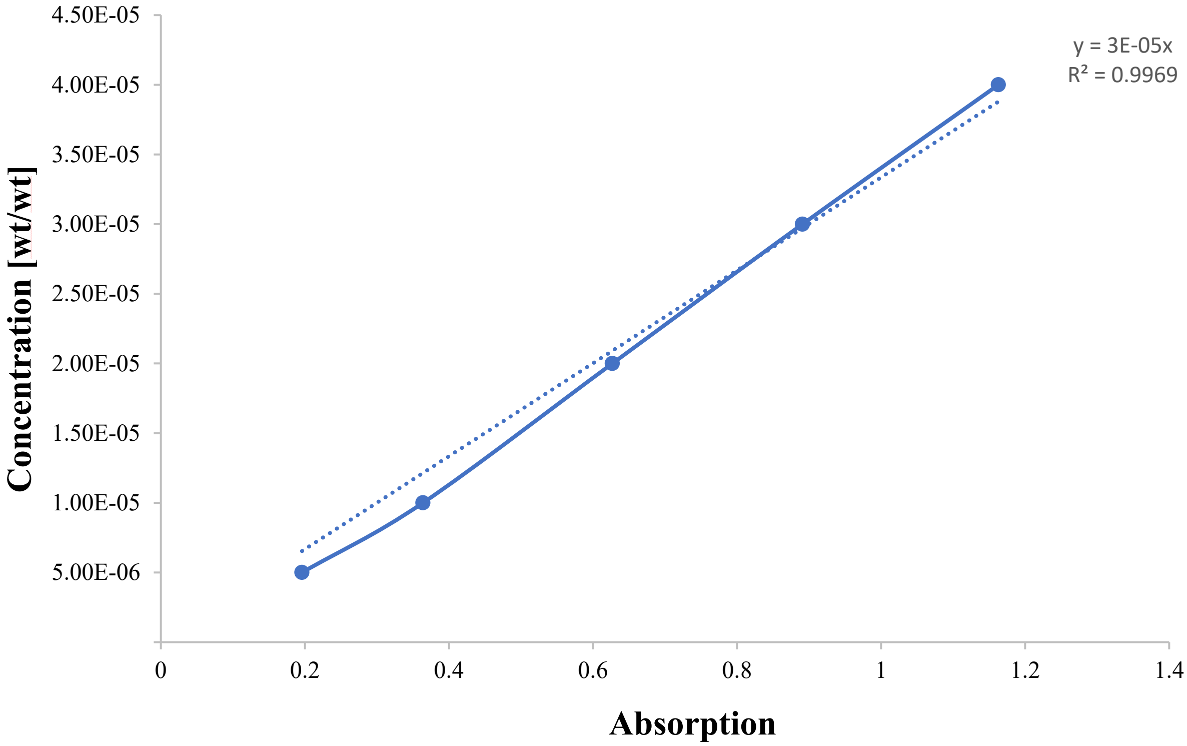

Figure S1. Allantoin Release Assessment - calibration curve.

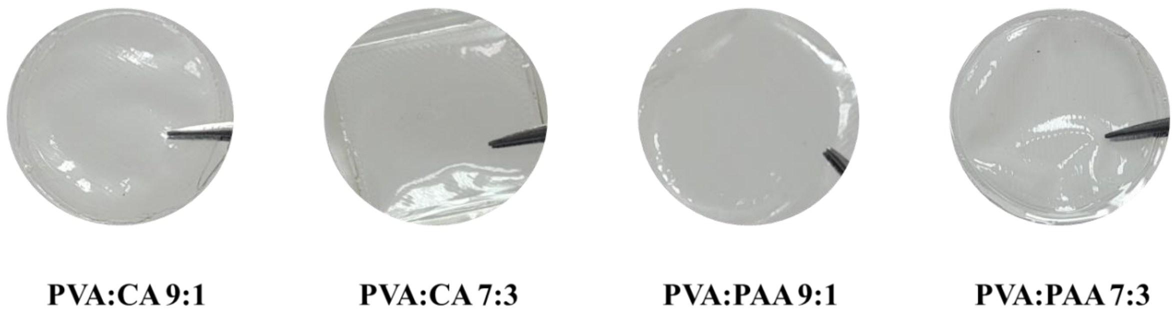

Figure S2: Images of the crosslinked thin films for different coating formulations.

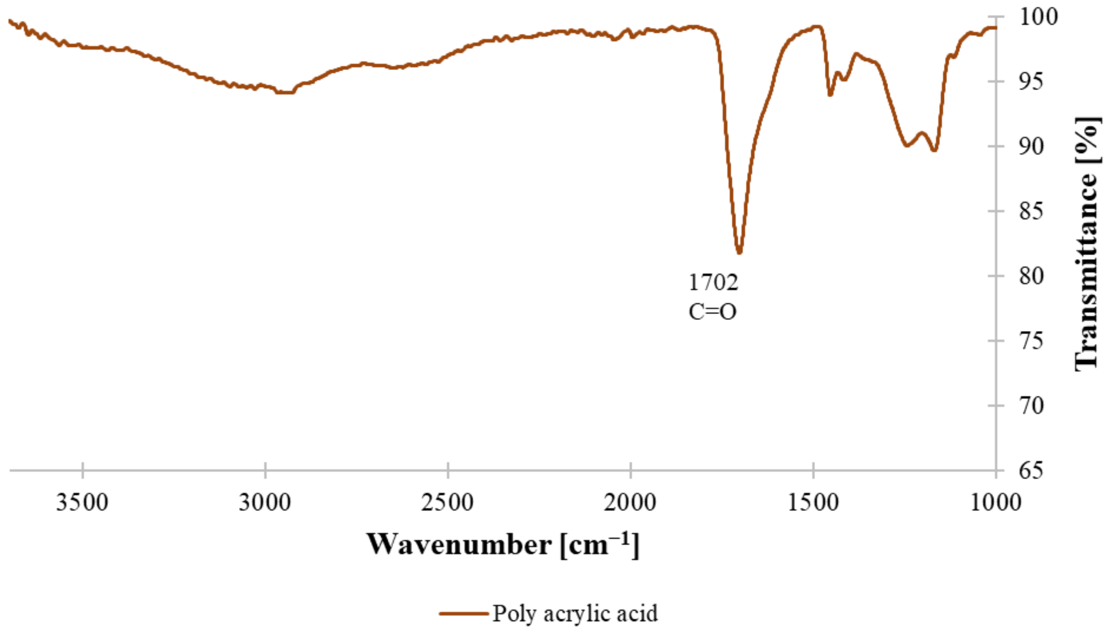

Figure S3. FTIR-ATR spectra of neat poly acrylic acid

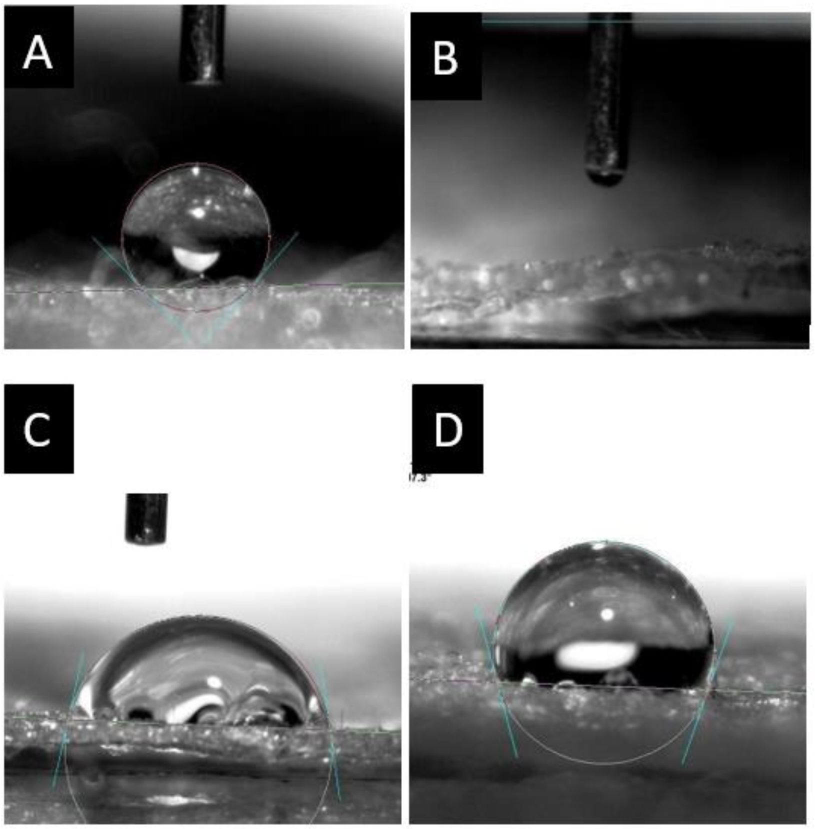

Figure S4. (A) polyethylene fabric neat; (B) polyethylene fabric coated with PVA and cured in 130℃ for 2-hour; (C) polyethylene fabric coated in PVA:CA 9:1; and (D) polyethylene fabric coated in PVA:CA 7:3.

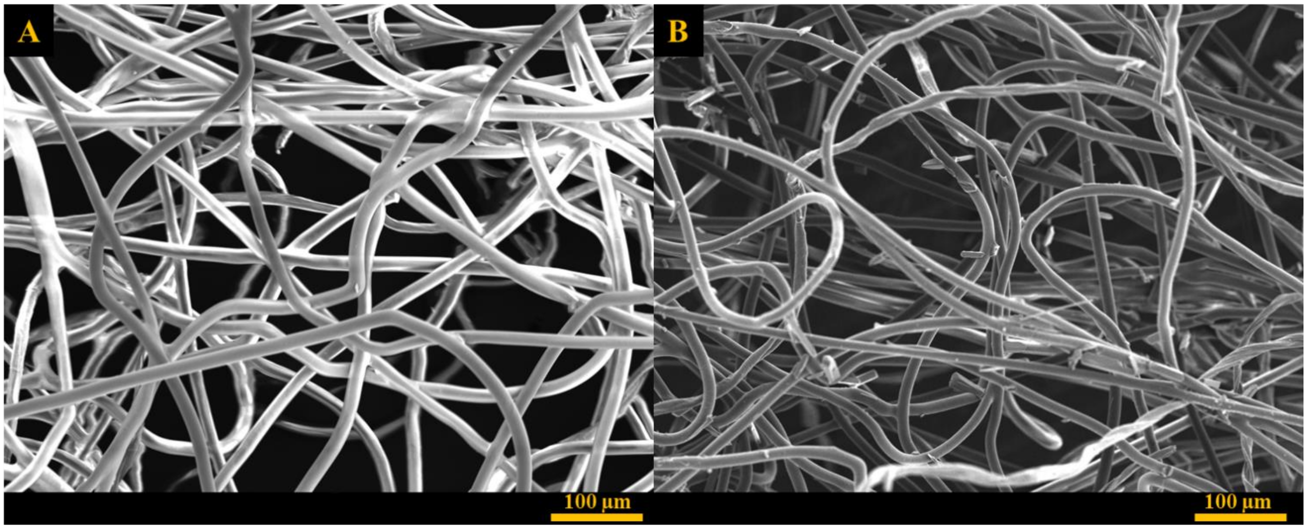

Figure S5. SEM image of polyethylene fabric (A) neat and (B) containing 15 wt% of allantoin.

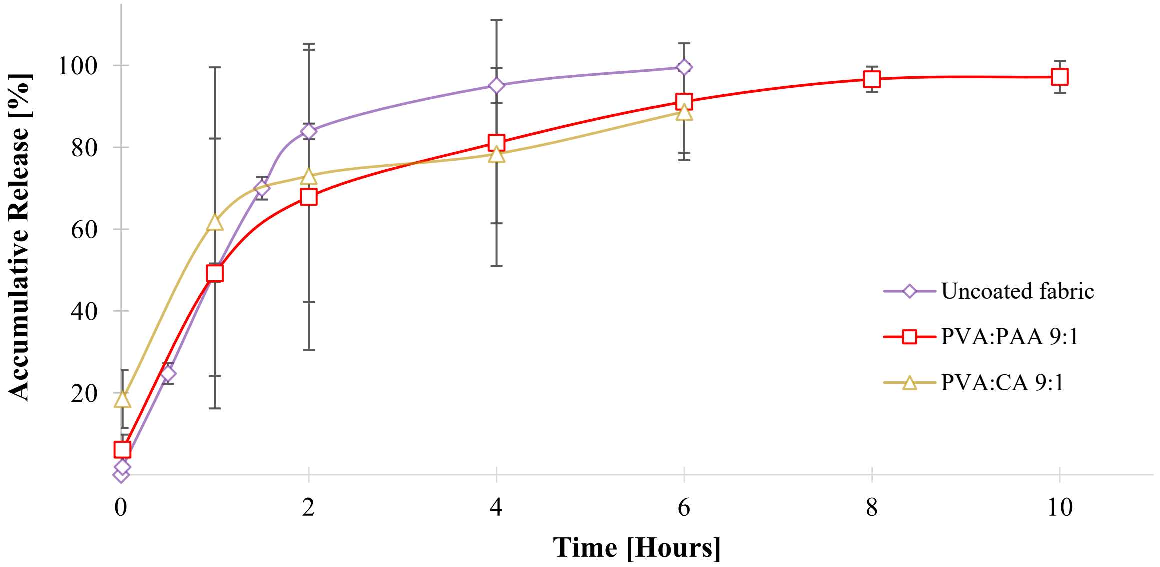

Figure S6. In—vitro drug release evaluation from PVA:PAA 9:1 and PVA:CA 9:1 formulation.

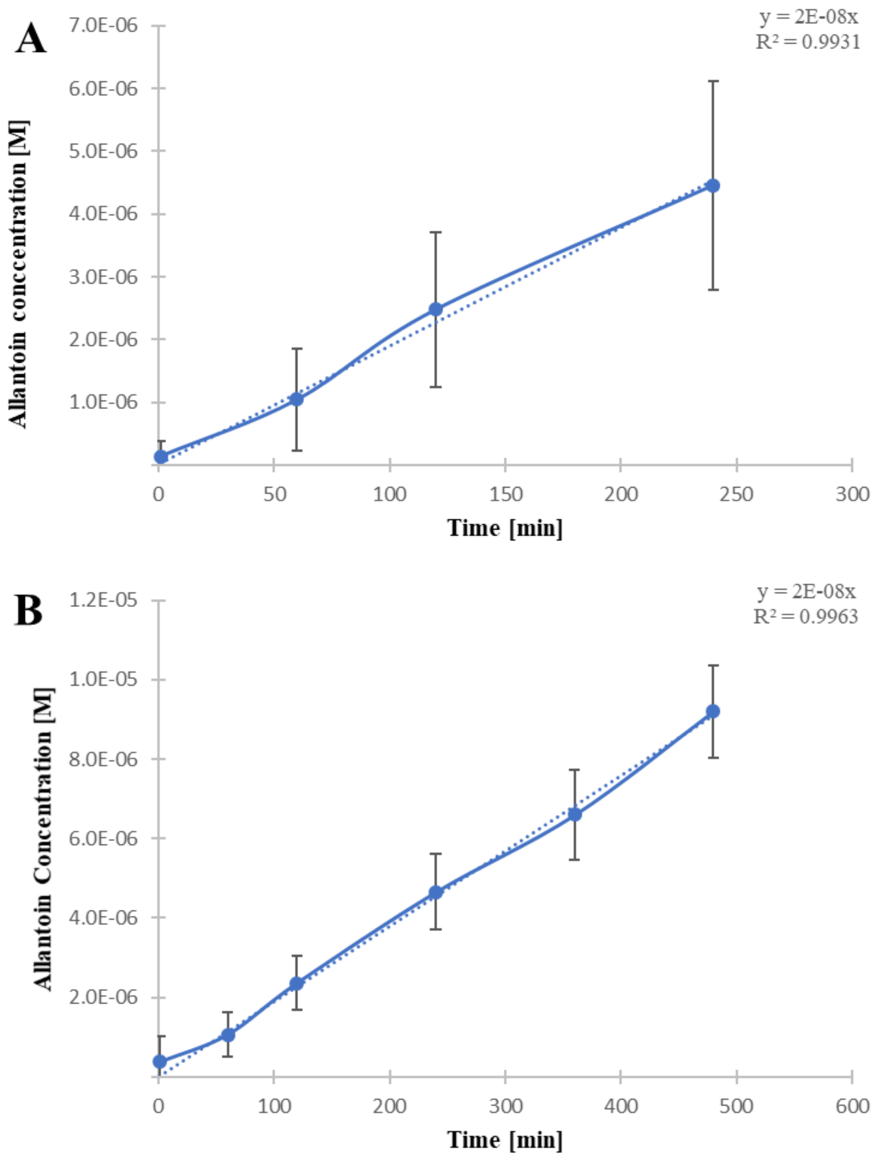

Figure S7. Comparison between (A) PVA:CA 7:3 and (B) PVA:PAA 7:3 to zero-order release kinetics.
